# The Book World of Medicine and Science

**Published:** 1901-02-16

**Authors:** 


### The Book World of Medicine and Science.

Hygiene and Public Health. By Louis Parkes, M.D., D.P.H.Lond.Univ., and Henry Kenwood, M.B., D.P.H., F.C.S. With illustrations. (London: H. K. Lewis. 1901. Price 12s.)

This work appears to be founded upon a previous one with the same title by Dr. Parkes, on which, however, it is a vast improvement, being practically a new book. It is so written as to meet the wants of those who are studying for the various Public Health diplomas, but will no doubt also be largely used by the profession at large, as well as by that growing number of laymen who, from their position as members of town and district councils, find it essential that they should know something about the sanitary questions which are constantly arising in the course of their public work. There is nothing particularly fresh in the general arrangement of the book. Water, refuse, air and ventilation, warming and lighting, soils and building sites, climate, exercise, food, the contagia, disinfection, statistics and law, follow in easy sequence and fairly well cover the field. Perhaps the part on law is a little compressed, and perhaps some who wish for more detailed information will be disappointed to find how general is the survey of all points dealing with chemistry and bacteriology. Nevertheless both these peculiarities make for readability, and although there is a noticeable absence of chemical formulæ and bacteriological details the authors have taken care that the results of the investigations made during recent years shall be thoroughly displayed. Indeed, they have produced an admirable and readable account of much recent work of a highly specialised nature. It is possible that a student confining his reading to this book (which, of course, he would never do, practical work being now essential) might be stumped at once by a culture of the most ordinary bacteria; yet he would probably know more about the bearing of bacteriology on public health questions than would a man who had devoted himself to laboratory study of individual micro-organisms; and to the majority that is the great thing. On the other hand, we cannot but feel that the almost entire exclusion of chemical considerations is a loss.

The many problems connected with water supply are adequately discussed, and we notice that, in considering the comparative advantages of "constant" and the "intermittent" service, it is mentioned that where the law compelling constant service is ostensibly complied with the companies in some cases insist on the insertion of a throttle of very small diameter (of one-eighth to one-sixteenth of an inch only) into the house communication pipe, "with the result that the water merely dribbles out of the house taps when they are full on;" a piece of trickery which ought to be dealt with not by individual householders but by the sanitary authorities of the districts wherever such an obvious evasion of the spirit of the law is attempted. In the chapter on the disposal of refuse considerable attention is given to the processes introduced of late years for the biological purification of sewage, and also to the problem of the sewage farm. As to the question whether sewage farms are productive of nuisance and injury to health it is stated that, although when badly managed and where the surface becomes sodden, and ponded sewage stagnates on it, it may become a nuisance, it has yet to be proved that such a farm can cause injury to health or produce disease; a pronouncement in regard to which we would only say that this depends upon what one calls disease. We may be permitted to doubt whether the condition of those who are continuously exposed to any "nuisance" arising from decomposing animal matter can properly be called health, however much they may maintain that they are well. When a sewage farm is so badly managed as to produce a nuisance, it certainly is a dangerous thing to attempt to draw the line and to say that it may not be injurious to health. A sewage farm when properly conducted ought not to be productive even of nuisance. The chapter dealing with contagia is full and complete, and will be found very useful by such non-medical readers as may refer to the book. We notice that in speaking of the different forms of apparatus for disinfecting by steam it is said that the steam should not be superheated. It is also truly enough stated that the amount of "superheat" which is generally given in practice is not sufficient to cause it to act very differently from saturated steam.

Take this book altogether we have no hesitation in saying that it is well arranged, thoroughly brought up to date, and in every way an excellent guide to the so-called science of public health, always, however, with this proviso, that a supplementary knowledge of chemistry and bacteriology, as indeed also of medicine, must be possessed by all who would wish to use it for examinational purposes.

---

### NOTES ON BOOKS.

The "Review of Reviews" office has just issued a little sixpenny book entitled "Stories of the Queen," which in a manner displays the history of the reign in a series of anecdotes. The idea is ingenious, and is fairly carried out The "stories," true or otherwise, will please many people.

The "Journal of Hygiene" (Cambridge University Press), the first number of which has just been issued, is a handsome quarterly which promises well to fill an important place in medical literature. There has been far too great a tendency of recent years, not perhaps to regard hygiene as a "popular science," but at any rate to treat hygienic subjects from a popular standpoint. This has no doubt been caused by the fact that most of the pronouncements of those officially engaged in sanitary work are of necessity addressed to non-medical sanitary authorities. The "Journal of Hygiene," however, if it goes on as it has begun, will do much to maintain the scientific bases on which hygienic work must always be founded, and to provide a professional medium for the publication of original work. The journal is edited by George H. F. Nuttall, M.D., Ph.D., in conjunction with John S. Haldane, M.D., F.R.S., and Arthur Newsholme, M.D., F.R.C.P.—a goodly trio. We wish it every success.

Although advertising and all its ways is a thing quite outside the professional life, we are not without a suspicion that it has a good deal to do with the success of medical as well as of other kinds of journalism, hence we turn with interest and indeed with curiosity to three noble volumes which at the present moment ornament our desk. Let us be alphabetical and begin with "Browne's 1901 Directory" (T. B. Browne), a handsome book and well arranged, giving all sorts of details about all sorts of newspapers. Among other peculiarities we notice that facsimiles of the title pages of many of the papers are given, and to make all quite clear and easy there are maps of many foreign countries. "Practical Advertising, 1901" (Mather and Crowther), is also a large and handsome volume, with long lists of newspapers, with a variety of useful information about them. "Willing's Press Guide" (James Willing, jun.) is a smaller but more business-like looking book. Its great feature is its A B C list, in which, although without tabulation, all the details required will be found. After this follow various classified lists, which will doubtless be found very useful.

